# Physiological Response of Stored Pomegranate Fruit Affected by Simulated Impact

**DOI:** 10.3390/foods12061122

**Published:** 2023-03-07

**Authors:** Pankaj B. Pathare, Mai Al-Dairi, Rashid Al-Yahyai, Adil Al-Mahdouri

**Affiliations:** 1 Department of Soils, Water and Agricultural Engineering, College of Agricultural & Marine Sciences, Sultan Qaboos University, Muscat 123, Oman; 2 Department of Plant Sciences, College of Agricultural & Marine Sciences, Sultan Qaboos University, Muscat 123, Oman

**Keywords:** impact energy, bruise susceptibility, pomegranate, respiration rate, color

## Abstract

Mechanical damage resulting from excessive impact force during handling and other postharvest operations from harvesting to consumption is a critical quality problem in fresh produce marketing. The study investigates the impact of bruise damage, storage temperature, and storage period on the physiological responses of Omani pomegranate fruit cultivar ‘Helow’. Fruits were subjected to low (45°; 1.18 J) and high (65°; 2.29 J) impact levels using a pendulum test by hitting the fruit on the cheek side. Bruised and non-bruised fruit were stored at 5 and 22 °C for 28 days. Bruise measurements, water loss per unit mass, water loss per surface area, firmness, fruit size measurements, geometric mean diameter, surface area, fruit volume, color parameters, respiration rate, and ethylene production rate were evaluated. Bruise area, bruise volume, and bruise susceptibility of damaged pomegranate fruit were increased as impact level, storage duration, and storage temperature increased. Pomegranates damaged at a high impact level and conditioned at 22 °C showed 20.39% weight loss on the last day of storage compared to the control and low-impact-bruised fruit. Firmness and geometric mean diameter were significantly (*p* < 0.05) reduced by bruising at a high impact level. Impact bruising level and storage temperature decreased lightness, yellowness, browning index, and increased redness over time. Furthermore, the respiration rate was five times higher in the non-bruised and low- and high-impact-injured fruit stored at 22 °C than that stored at 5 °C. The ethylene production rate recorded its highest value on day 21 in high-level-impact-bruised pomegranate fruit. The bruise susceptibility was strongly correlated with the majority of the studied parameters. This study can confirm that bruising can affect not only the visual quality characteristics but also the physiological attributes of pomegranate fruit; therefore, much care is required to preserve fresh produce and avoid any mechanical damage and losses during postharvest handling.

## 1. Introduction

Pomegranates have become a popular commercially grown fruit in many regions of the world. A high number of commercial pomegranate trees orchards are grown in different parts of the world [1]. The consumption of pomegranates has shown a remarkable increase due to their exceptional nutritional and sensory attributes linked with various medicinal advantages attributed to the fruit’s healthy substance helping to provide high antioxidant and phytonutrient capacity [2]. However, using inappropriate handling equipment in the supply chain can make pomegranate fruit prone to external mechanical forces that finally lead to bruising [3].

Mechanical damage in fresh produce is the leading cause of postharvest quality damage and losses during handling and other postharvest operations [4]. Mechanical damage, including bruising in fresh produce during handling and transportation, is attributed to different types of forces such as compression, abrasion, impact, and cutting [5]. Bruising results from action due to severe external forces on the fresh produce surface when fruit hits other fruit or another rigid surface/body during handling [6]. Bruising is known as a failure observed in the subcutaneous tissue of the impacted fruit without rupturing the skin. Discoloration can be observed in the damaged tissues, which indicates the injured spot [7].

Previous research has shown that the occurrence of bruising can affect the exterior fruit attributes, interior quality deterioration, physiological process alterations, and increase postharvest decay [8]. Besides, bruising can reduce the weight of different fruit and vegetables, thus reducing their market value [9]. Moreover, it modifies the metabolic and physiological processes, resulting in faster ripening, browning, and other quality and economic losses [10]. Bruising causes softening and accelerates the changes in color attributes of bruised fruit. The negative consequences of bruising can reduce the shelf life of produce. Bruising is considered a risk for fungal and bacterial infection [11]. Studies in bruising have shown that the respiration rate increased with increasing impact levels. In addition, it can reduce firmness and cause different changes in the lycopene of tomatoes [12].The intensity of a bruise can be expressed as bruise diameter, area, and susceptibility. The diameter and depth of the bruise are the primary direct measurements utilized to define the bruise size [10]. Several methods are used to replicate fruit bruising in the laboratory and test the effect of impact on different agricultural products. The most common methods used are the drop test [13,14] and the pendulum method [15]. These methods are mainly structured to simulate various types of dynamic loading that occur during the time of harvesting operations. The present study concentrated on the applied impact force, which is the most common cause of loading [3], particularly in pomegranate fruit. Therefore, an impact test involving dropping a pendulum arm with a particular weight from the desired angle into the fruit is one of the commonly known techniques and has been applied to study bruising measurements of different fruit such as apples [16], pears [15], nectarines [17], and grain (pea pods) [18]. There is no study evaluating the influence of impact damage using a pendulum impact test during storage on the physiological attributes of Omani pomegranates. Therefore, this study aims to assess the effect of two different impact levels on the bruising magnitude and the quality characteristics of the pomegranate cultivar ‘Helow’ stored at two storage conditions (5 and 22 °C) for 28 days (water loss, firmness, size, and color) and 56 d (respiration and ethylene production rate) of storage.

## 2. Materials and Methods

### 2.1. Fruit Selection and Preparation

Pomegranates (*Punica granatum* L.) of the cultivar ‘Helow’ (sweet) were harvested manually from farms located in Al-Jabal Al-Akhdar, Ad-Dakhliyah Governate, Oman. Pomegranates were packaged in cardboard boxes and delivered to Postharvest Technology Laboratory, Sultan Qaboos University, Oman. The duration of transportation was about two hours. On arrival, pomegranate fruits were sorted to ensure color (lightness (L*) = 55.90 ± 3.10, redness (a*) = −3.63 ± 0.95, and yellowness (b*) = 53.17 ± 2.78), weight (456  ±  0.033 g), and size (length (*L*) = 90.555 ± 0.162 mm, width (*W*) = 91.47 ± 0.240 mm, and thickness (*T*) = 94.265 ±  0.187 mm) uniformity and that the samples were free from surface blemishes, cracks, and defects. A total of 75 pomegranate samples were used for the present study.

### 2.2. Fruit Impact Bruising (Pendulum Test) and Storage

**Figure 1:**
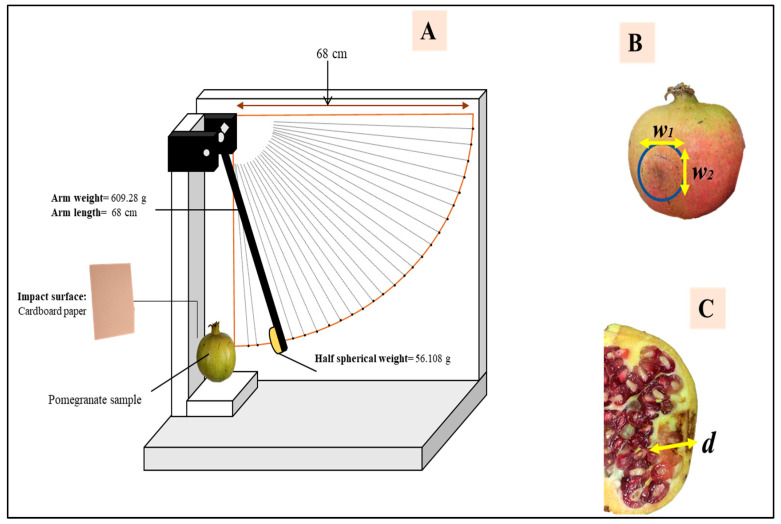
The schematic diagram of the experimental setup (**A**), bruised pomegranate fruit with bruise diameter (*w*_1_ and *w*_2_) (**B**), and sliced pomegranate fruit showing the bruise depth (*d*) (**C**).

A pendulum system was designed to produce bruises in pomegranate fruit (Figure 1A). Unlike the free drop of mass (weight) through a hollow-guided tube, the pendulum concept permits better observation and control of the falling weight during testing [19]. The size of the steel pendulum arm (mass = 609.25 g, length = 68 cm) was carefully selected to trigger the tested fruit. A half-spherical weight (56.10 g) is connected to the pendulum arm to damage the pomegranate fruit. This test was conducted by raising the pendulum arm from a specific angle and then dropping it once to hit/damage the tested fruit with the half-spherical weight. To generate various energy levels, the pomegranates were divided into three groups. The first and second groups included pomegranate fruit bruised (cheek-side) from angles of 65° and 45° that represent the high and low impact levels, respectively. The third group was considered a control (without damage). Each group includes 24 (*n* = 24) pomegranate fruit. After the initial rebound, the arm was caught by hand to avoid repeated impacts.

Under these conditions, impact energy (*E_i_*-J) (Equation (1)) was determined assuming that the fruit absorbed all the energy of the dropped mass [19]:

(1) $$E_{i}=mgh_{1}$$

To calculate the actual energy absorbed (*E_a_*) by the pomegranate fruit that results in the measured bruise damage, it is essential to estimate the equivalent rebound height (*h*_2_-cm) at the maximum rebound. The equivalent rebound height (*h*_2_-cm) was used to calculate the rebound energy (*E_r_*-J) as follows (Equation (2)) [19]:

(2) $$E_{r}=mgh_{2}$$

The graduated scale on the whiteboard was used to record the actual rebound angle. In addition, a camera (Model: EOS FF0D, Canon Inc., Tokyo, Japan) was utilized to record the accurate readings of the rebound angles (equivalent rebound heights). The absorbed energy (*E_a_*-J) was determined from the difference between energy at first impact and rebound, as shown in Equation (3) [17]:

(3) $$E_{a}=mg(h_{1}-h_{2})$$

where *m* is the mass of the steel ball (kg), *g* is the gravitational constant (9.81 ms^−2^), *h*_1_ is the equivalent drop height (cm), *h*_2_ is the equivalent rebound height (cm), *E_i_* is the impact energy (J), *E_r_* is the rebound energy (J), and *E_a_* is the absorbed energy (J).

The bruise size was measured by slicing the center of the damaged area (marked) of each pomegranate fruit. The bruise damage of the sliced fruit was estimated by the presence of apparently damaged tissues, which were visibly distinguishable from other unbruised (undamaged) parts of the same pomegranate fruit. Major (*w*_1_) and minor (*w*_2_) diameters (Figure 1B) and bruise depth (*d*) (Figure 1C) of the elliptical bruise shape were identified using a digital caliper (Model: Mitutoyo, Mitutoyo Corp., Kawasaki, Japan). Results of bruise damage size on pomegranate fruit were expressed as bruise area (*BA*-mm^2^) and bruise volume (*BV*-mm^3^) (Equations (4) and (5)). The bruise susceptibility (*BS*-mm^3^/J), which is the ratio of the *BV* to the energy absorbed (*E_a_*) during the pendulum experiment impact, was also measured using Equation (6). For possible reduction of further implications of fruit mass on resulting *BS*, specific bruise susceptibility (*SBS*-mm^3^ J^−1^ g^−1^), also known as bruise sensitivity index, was identified by using Equation (7) below [14].

(4) $$BA=\frac{\pi }{4}w_{1}\;w_{2}$$

(5) $$BV=\frac{\pi d}{24}\left(3w_{1}w_{2}+4d^{2}\right)$$

(6) $$BS=\frac{BV}{E_{a}}$$

(7) $$SBS=\frac{BS}{m_{f}}$$

where *m_f_* is the pomegranate fruit mass.

After the impact test, each group (high- and low-impact-bruised fruit groups and the control group) was divided equally into two sets and stored at cold temperature (5 ± 1 °C; 95 ± 5% RH) and ambient temperature (22 ± 1 °C; 80 ± 5% RH) to investigate the effect of bruising and storage temperature on pomegranate fruit, and different physiological attributes (fruit size, weight/water loss, firmness, color, respiration rate, and ethylene production rate) for 28 days, as described in Section 2.3. All measurements were taken after 3, 7, 14, 21, and 28 days. However, two more measurements (readings) were taken for both the respiration rate and ethylene production rate. A total of 3 pomegranate fruits were analyzed for day-0 analysis.

### 2.3. Quality Measurements

#### 2.3.1. Water Loss

Water loss was determined with respect to the pomegranate fruit weight (*WL*%) (Equation (8)) and in terms of the unit surface area (*WL_A_*-g cm^2^) (Equation (9)) [20]. Pomegranate fruit mass was determined using an electric weight balance (Model: GX-4000, Japan) with an accuracy of ±0.01 g.

(8) $$WL\;\%=\frac{\left(W_{i}-W_{t}\right)}{W_{i}}\times 100$$

(9) $$WL_{A}=\frac{\left(W_{i}-W_{t}\right)}{A_{s}}$$

where *W_i_* is the initial weight on day 0 and *W_t_* is the recorded fruit weight after storage day.

#### 2.3.2. Firmness

Two opposite sides of each pomegranate fruit peel (non-bruised parts) were used to record firmness (N) by using a digital fruit firmness tester (Model: FHP-803, L.L.C., USA) with an 8 mm diameter probe. A total of 4 readings were taken from two fruit of each group (high- and low-impact-bruised fruit groups and the control group) per storage condition, per day.

#### 2.3.3. Geometric Mean Diameter, Surface Area, and Fruit Volume

Length (*L*-mm), width (*W*-mm), and thickness (*T*-mm) of pomegranate fruit were determined by three linear dimensions. Each pomegranate fruit’s length (*L*) was determined at the longitudinal perimeter (without calyx). The width (*W*) and thickness (*T*) were taken at the equatorial perimeter [20]. The measurements were recorded using a digital caliper and the geometric mean diameter (*Dg*-mm) was calculated using Equation (10). The surface area (*A_s_*-mm^2^) was calculated using the *Dg* and pi presented in Equation (11). In addition, the volume (*V*-mm^3^) of pomegranate fruit was calculated from the *LWT* parameters and pi number using Equation (12) [21].

(10) $$Dg={\left(LWT\right)}^{0.3333}$$

(11) $$A_{s}=\pi {\left(Dg\right)}^{2}$$

(12) V=πLWT/6

#### 2.3.4. Color

Pomegranate fruit peel color evaluation was conducted using a computer vision system (CVS) explained by Al-Dairi et al. [22]. In addition, the ImageJ software (v. 1.53, National Institute of Health, Bethesda, MD, USA) was applied to process and analyze all acquired red, green, and blue values (RGB) from the system. Later, RGB values were transferred to CIEL*a*b* color space. L*, a*, and b* for lightness, redness/greenness, and yellowness/blueness, respectively, were measured on bruised and non-bruised fruit (excluding the bruised/marked area) for each sample per group stored at two different conditions. A total of 60 readings were taken per day (10 per group). Chroma (C*) and hue° (h*), which describe the color intensity and purity, respectively, were calculated (Equations (13) and (14)). The total color difference (*TCD*) and browning index (*BI*) given in Equations (15) and (16) were also measured.

(13) $$C*=\sqrt{a{*}^{2}+b{*}^{2}}$$

(14) $$H*=\tan^{-1}\left(\frac{\;b*}{a*}\right)$$

(15) $$TCD=\sqrt{\Delta {a*}^{2}+\Delta b^{2}+\Delta {L*}^{2}}$$

(16) $$BI=100\times (X-0.31/0.17)\;\mathrm{Where}\;X=\frac{\left(a*+1.75L*\right)a*}{\left(5.645L+a*-3.012b*\right)}$$

#### 2.3.5. Respiration Rate and Ethylene Production Rate

Respiration rate and ethylene production rate of bruised and non-bruised (control) pomegranate fruit were measured after 3, 7, 14, 21, 28, 48, and 56 d of the impact experiment using the method of the closed system described earlier by Hussein et al. [9] and Pathare and Al-Dairi [23]. For each group, two plastic boxes (2.3 L) containing one pomegranate fruit were used to measure the respiration (CO_2_) rate and ethylene (C_2_H_4_) production rate. O_2_/CO_2_ analyzer (Model: 90 2D, Quantek Instruments, Inc., Grafton, Australia) and ethylene detector (Model: SCS 56, Fricaval89, Valencia, Spain) were used to measure respiration rate and ethylene production rate, respectively. Respiration and ethylene production rates were calculated according to Castellanos et al. [24].

### 2.4. Statistical Analysis

The results were statistically analyzed using SPSS 20.0 (International Business Machine Crop., Armonk, NY, USA) software. Analysis of variance (ANOVA) was implemented to assess the influence of three investigated factors (A: impact damage level; B: storage temperature; and C: storage duration) and the factors’ interaction with the physiological attributes of pomegranate fruit at a 5% level of significance. GraphPad Prism software 9.3.1 (GraphPad Software, Inc., San Diego, CA, USA) was applied for graph construction. In addition, the Pearson correlation coefficient was calculated to evaluate the relationship between pomegranate fruit quality parameters. Minitab statistical software 21.2 (State College, PA, USA) was also used to perform principal component analysis.

## 3. Results and Discussion

### 3.1. Measurements Related to Bruising

**Figure 2:**
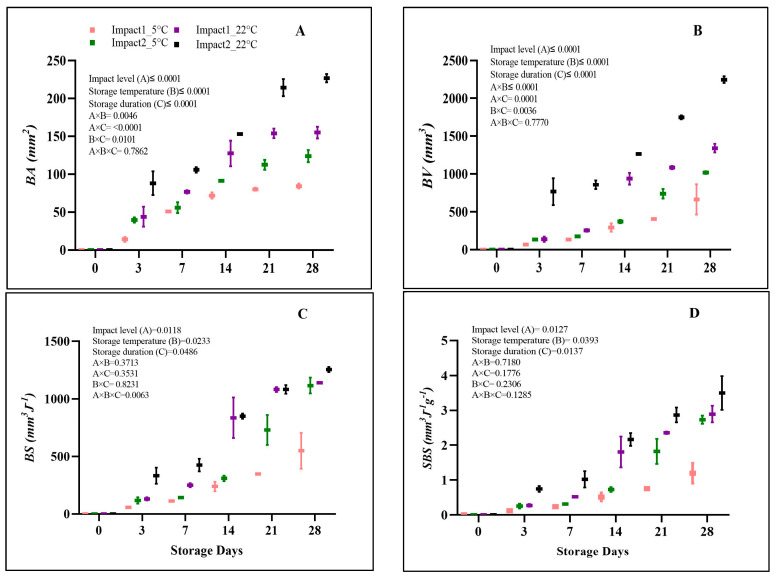
Bruise area (**A**), volume (**B**), susceptibility (**C**), and specific bruise susceptibility (**D**) of pomegranate fruit impacted at different impact angles (45°; 1.18 J and 65°; 2.29 J) during 28 days at 5 °C and 22 °C storage conditions. The data are presented using a whisker plot. The total number of readings per day per treatment is 2.

Table 1 shows the results of the impact, rebound, and absorbed energies generated from the pendulum system during the dropping of a 56.10 g weight on pomegranate fruit from different angles (45° and 65°). Increasing the drop angle from 45° to 65° increased the energy absorbed by the fruit during impact. Figure 2 presents the overall results of bruising measurements. Impact damage level, storage temperature, and duration statistically (*p* < 0.05) affected both *BA* and *BV* of damaged pomegranate fruit (Figure 2A,B). The findings showed that *BA* and *BV* increased significantly from the lower impact level (1.18 J) to the higher (2.29 J) impact level during the storage period. Pomegranate fruit stored at 22 °C and bruised at the highest impact level showed the highest *BA* (226.61 mm^2^) and *BV* (2447.51 mm^3^) values on the last day of storage. The lowest *BA* and *BV* values were recorded in the pomegranate fruit bruised at the low-impact level and stored at 5 °C with 88.10 mm^2^ and 663.90 mm^3^, respectively. These results are in accordance with the findings recorded by Shafie et al. [3], where the impact energy level was the main parameter identifying the *BV* in bruised pomegranate fruit. Later, Shafie et al. [25] confirmed that the *BV* of pomegranate fruit was primarily proportional to the impact energy and the drop height using different impact surfaces. Tabatabaekoloor [26] stated that as the fruit dropped from the highest level of damage, more potential energy was generated, potentially expediting the content intensity, and resulting in increased *BA*.

Similarly, Hussein et al. [14] reported a significant increment in *BA* across all pomegranate cultivars when the impact level increased. They recorded a 39.1% and 18.6% increase in *BA* after doubling the drop height from 20 to 40 cm and 40 to 60 cm, respectively. Moreover, Pathare and Al-Dairi [7] revealed a significant relationship between the main factors (drop height level and storage condition) and the resulting values of *BV* and *BA* of pears during 14 days of the storage period.

The *BS* was influenced by damage level (*p* = 0.0118), storage temperature (*p* = 0.0233), and storage duration (*p* = 0.0486) (Figure 2C). Alterations in *BS* with increasing impact levels were consistent across all storage conditions. Increasing the level of impact from 1.18 J (low) to 2.29 J (high) intensified the *BS* of the bruised pomegranate by 10.14%. The highest *BS* values were observed on pomegranate stored at 22 °C and damaged at the highest impact level (1254.93 mm^3^J^−1^), followed by those impacted at the lowest impact level (1139.34 mm^3^J^−1^) after 28 days of storage. *BS* values of pomegranate fruit bruised at low and high impact levels were 549.27 mm^3^J^−1^ and 1116.11 mm^3^J^−1^, respectively, on day 28 of storage at 5 °C. Ahmadi [27] emphasized that storage temperature influences cell wall strength and viscosity. Ahmadi et al. [28] suggested that storage at high-temperature conditions can boost the incidence of bruising in fresh fruit due to the active status of enzymes, hence resulting in stiffness and cell wall degradation. Similarly, Pathare and Al-Dairi [12] found a high bruise occurrence in tomatoes stored at 22 °C compared to those stored at 10 °C. Bugaud et al. [29] observed lower *BS* on bruised bananas stored in low-temperature conditions. In addition, this study revealed that increasing storage duration increased bruising. Azadbakht et al. [15] found that bruising increased by 0.21% and 47.36% on days 5 and 15, respectively.

Figure 2D shows the results of bruise sensitivity tests which are presented as specific bruise susceptibility (*SBS*). There was a significant effect of the level of impact (*p* = 0.0127), temperature (*p* = 0.0393), and duration (*p* = 0.0137) on *SBS* values of the bruised pomegranates. The *SBS* gradually increased with impact level at both storage conditions during 28 days of storage. Overall, the *SBS* value was the highest for pomegranates bruised following the highest impact (dropped from an angle of 65°) under ambient (22 °C) temperature conditions with 3.49 mm^3^J^−1^g^−1^, followed by pomegranate fruit bruised at the lowest impact level (dropped from an angle of 45°) with 2.89 mm^3^J^−1^g^−1^ after 28 days of storage. Pomegranate fruit stored at 5 °C for 28 days showed the lowest value of *SBS* with 1.19 mm^3^J^−1^g^−1^. The study can suggest that pomegranate fruit stored at ambient (22 °C) temperature conditions after being damaged at a high impact level could be the most sensitive to bruising after a prolonged storage duration.

### 3.2. Quality Attributes Changes

#### 3.2.1. Water Loss and Firmness

**Figure 3:**
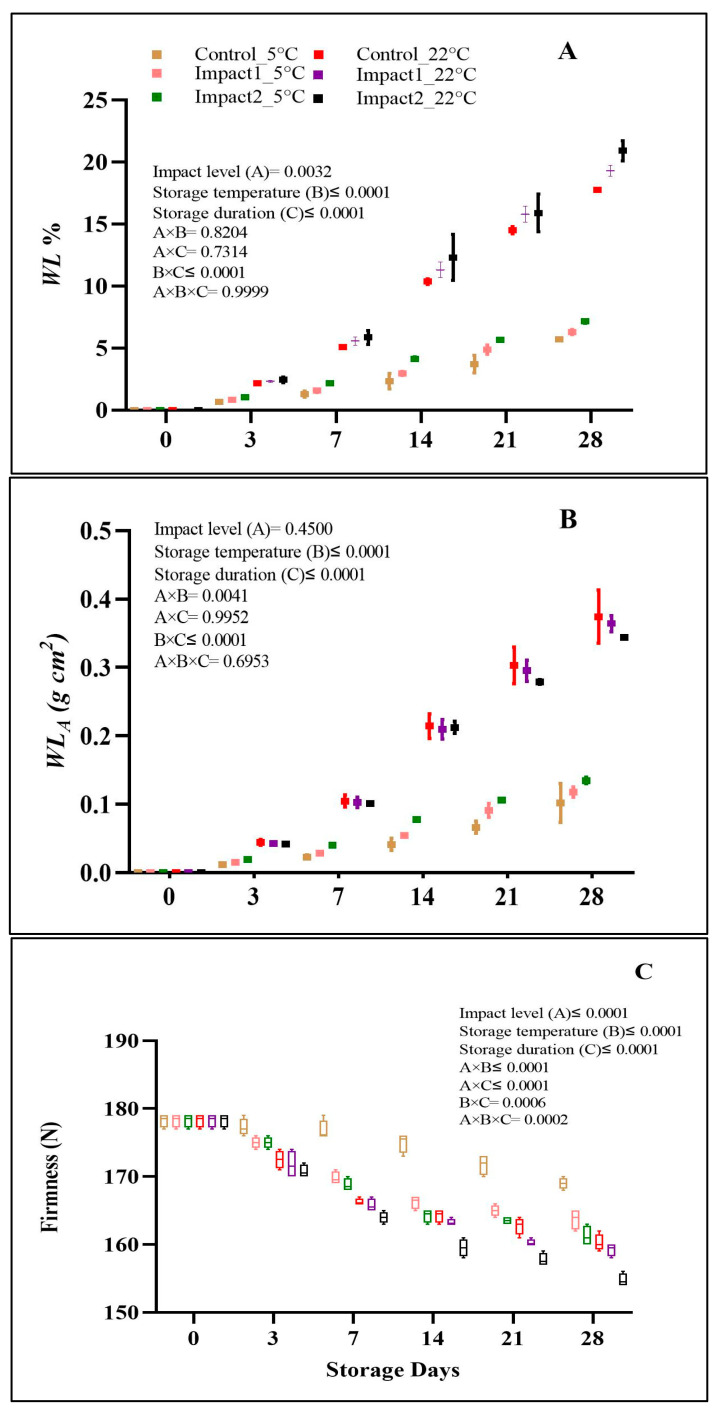
Weight loss % (**A**), water loss per unit surface area (**B**), and firmness (**C**) of non-bruised, low- (45°; 1.18 J), and high- (65°; 2.29 J) impact-bruised fruit during 28 days at 5 °C and 22 °C storage conditions. The data are presented using a whisker plot. The total readings per day per treatment are 2 for WL% and *WL_A_* and 4 for firmness.

The water loss per unit fruit mass (*WL%*) and per unit surface area (*WL_A_*) profiles of bruised and non-bruised pomegranate fruit stored at 5 and 22 °C for 28 days are presented in Figure 3A,B. The analysis of variance showed that the impact level, storage temperature, and storage duration significantly (*p* < 0.05) influenced *WL%* (Figure 3A). After 28 days of storage at 22 °C, the highest *WL%* was observed in pomegranate fruit bruised at the highest impact level (20.39%), followed by those impacted at the lowest impact level (19.29%) and the non-bruised (control) fruit (17.74%). At the end of 28 days of storage at a cold temperature (5 °C), the average *WL%* measured in pomegranate fruit impacted at high and low levels was 7.14 and 6.28%, respectively. A lower % of *WL* was observed in the non-bruised control fruit with 5.70% under low-temperature storage conditions on day 28. The results of water loss per unit surface area (*WL_A_*) did not follow the trend of measured *WL*% (Figure 3B). The effects of storage duration and temperature on *WL_A_* were significant (*p* < 0.05). However, no pronounced effect of impact level on *WL_A_* (*p* > 0.05) could be observed. The *WL_A_* values increased with temperature and storage time, in non-bruised and bruised pomegranate fruit. The expectation was confirmed in ambient-conditioned pomegranate fruit. For instance, the non-bruised fruit showed the highest *WL_A_* (0.37 g cm^2^) followed by low- (0.36 g cm^2^) and high-level (0.34 g cm^2^) impacted pomegranate fruit. The *WL_A_* of pomegranate fruit stored at 5 °C was lower than that of fruit stored at ambient conditions. Generally, the findings from the current study have confirmed that bruise damage could expedite the physiological *WL* and pomegranate fruit senescence during storage.

In terms of storage conditions, Hussein et al. [14] and Fawole and Opara [1] suggested that storage at 5 °C reduced the moisture content loss of bruised and non-bruised fruit resulting in a low increment in *WL%* of the fruit during the storage. This might be because of the metabolic activity reduction rate at low temperatures. Ambaw et al. [30] revealed that *WL%* in pomegranates during storage increased due to the fruit peel’s high porosity, which enables the movement of free vapor. Hussein et al. [14] found that the *WL%* on bruised pomegranate fruit stored at low storage was eight-fold lower than that of fruit stored under ambient conditions. As revealed in the present study, the weight loss increased as bruising increased. Tissue damage caused by bruising can permit the interchange of atmospheric gases, resulting in increasing respiration and transpiration rates [13], which showed in some symptoms of wilting and shriveling in all fruit samples stored at 22 °C starting from day 21.

The results from this study showed that the firmness of the bruised and the non-bruised pomegranate fruit was significantly (*p* ≤ 0.0001) dependent on impact level, storage duration, and storage temperature (Figure 3C). Pomegranate fruit firmness values were reduced by increasing all investigated factors. Both temperature and impact were highly pronounced in the bruised and the control pomegranate fruit. On the last d of storage, the firmness was reduced by 5.1, 8.13, and 9.51% in the control, low-, and high-impact-bruised fruit stored at 5 °C. However, the reduction was higher in the pomegranate stored at 22 °C, where the reduction % reached 10.09, 10.65, and 13.18% for the control, low-, and high-impact-bruised fruit, respectively, on the last day of the experiment. Generally, bruising decreased the firmness status of pomegranate fruit, particularly under ambient storage conditions during the prolonged storage period. Similar findings were observed by Azadbakht et al. [15] and Pathare and Al-Dairi [7].

Regarding storage conditions, Arendayse et al. [31] revealed that increasing storage temperature and storage duration could reduce the firmness of pomegranate fruit, probably due to the decline in the cell wall integrity of the pomegranate fruit peel. In addition, the chilling injuries produced on fruit at low temperatures could be the main reason for firmness and cell wall strength reduction in bruised and non-bruised pomegranate fruit at 5 °C. The study of Pathare et al. [13] recorded a significant decrease in the firmness of pear fruit when the impact energy increased from 0.129 J (low) to 0.38 J (high).

#### 3.2.2. Size Measurements, Geometric Mean Diameters (Dg), Surface Area (A_S_), and Fruit Volume (V)

Table S1 shows the size dimension measurement reduction % of the bruised and the non-bruised pomegranates stored at two storage conditions for 28 days. The analysis of variance showed that all the studied factors (impact level, storage duration, and storage temperature) significantly influenced (*p* < 0.05) the length (*L*) and thickness (*T*) of the investigated fruit. The widths (*W*) of the bruised and the non-bruised pomegranate fruit were affected statistically (*p* < 0.05) by storage temperature and duration but not by the impact level (*p* > 0.05). The % of loss in *LWT* gradually increased as all investigated factors increased. For instance, pomegranates bruised at a higher level and stored at 22 °C showed a higher % of loss on *LWT* with 2.57, 2.36, and 2.38%, respectively. Generally, the fruit dimension (*LWT*) is the main factor influencing the overall geometric mean diameter (*Dg*), surface area (*A_S_*), and fruit volume (*V*) [32].

**Figure 4:**
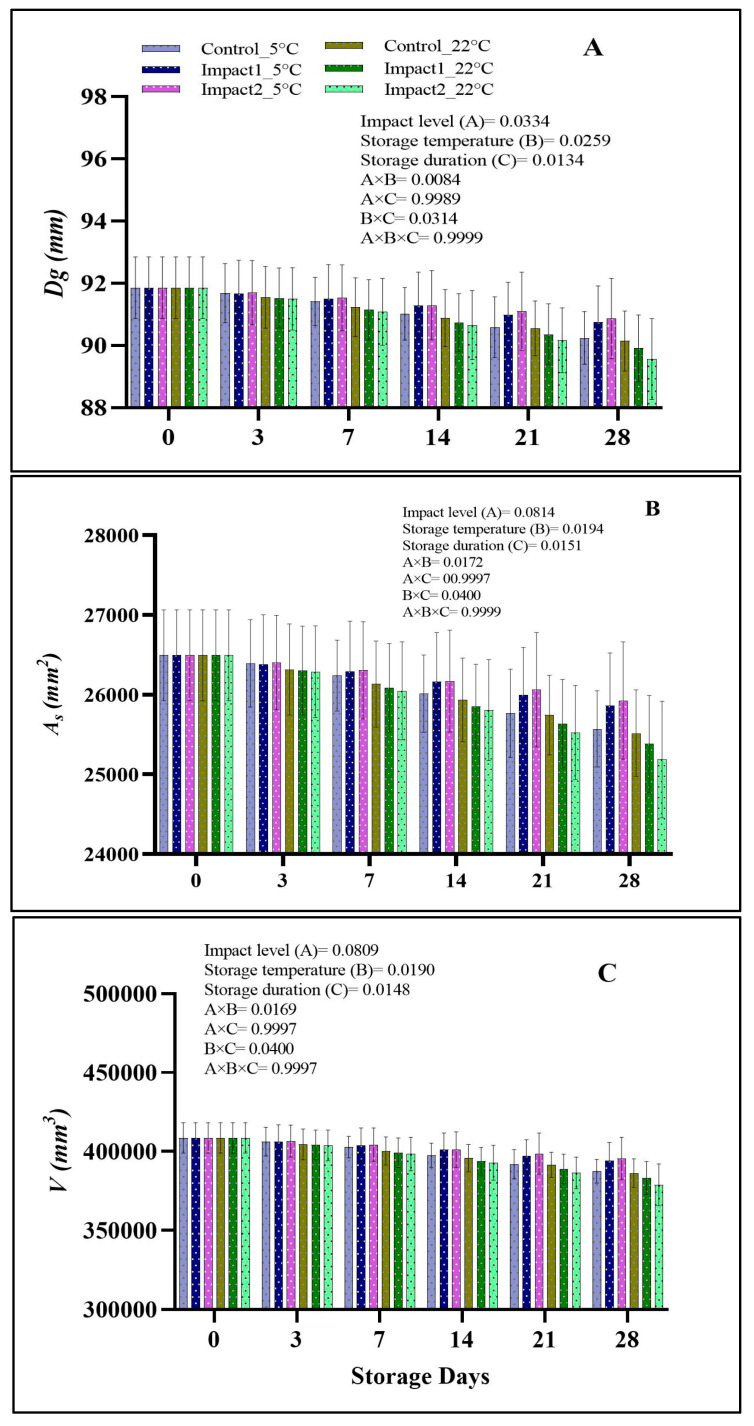
Geometric mean diameters (**A**), surface area (**B**), and fruit volume (**C**) of non-bruised, low—(45°; 1.18 J), and high—(65°; 2.29 J) impact-bruised fruit during 28 days at 5 °C and 22 °C storage conditions. The data are presented as the mean ± SD of 2 readings per treatment.

There was a significant effect of impact damage (*p* = 0.0334), storage condition (*p* = 0.0259), storage time (*p* = 0.0134), and their interaction on the *Dg* values of control and damaged pomegranate fruit (Figure 4A). The values of *A_S_* and *V* were highly influenced (*p* < 0.05) by storage temperature and duration with no pronounced effect with impact level (*p* > 0.05) (Figure 4B,C). At ambient (22 °C) storage conditions, pomegranate fruit bruised at higher (*E_a_* = 2.28 J) and lower (*E_a_* = 1.18 J) impact levels exhibited *Dg* values of 89.55 and 89.90 mm after 28 days of storage compared to the non-bruised fruit with 90.14. By contrast, the lowest value of *Dg* at the 5 °C storage condition was detected in the non-bruised (control) (90.24 mm) pomegranate fruit, followed by the bruised pomegranates at lower (90.75 mm) and higher (90.86 mm) impact levels, respectively, after 28 days of storage. A similar scenario was observed for the *A_S_* and *V* values of both bruised and non-bruised fruit at both storage conditions. The reduction observed in *LWT*, *Dg*, *A_S_*, and *V* during storage for all samples is attributed to water loss [33]. In addition, the bruising caused by external factors such as impact can cause damage to the internal cells, which leads to increased respiration rate and enzymatic activity due to vibration, as observed by Dagdelen and Aday [21].

#### 3.2.3. Peel Color

**Figure 5:**
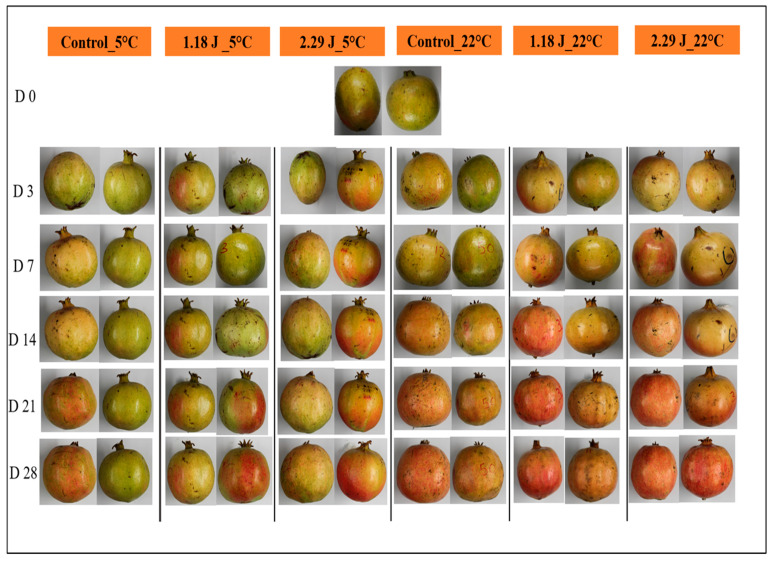
The overall color change of pomegranate fruits groups during 28 days (D) of storage.

**Figure 6:**
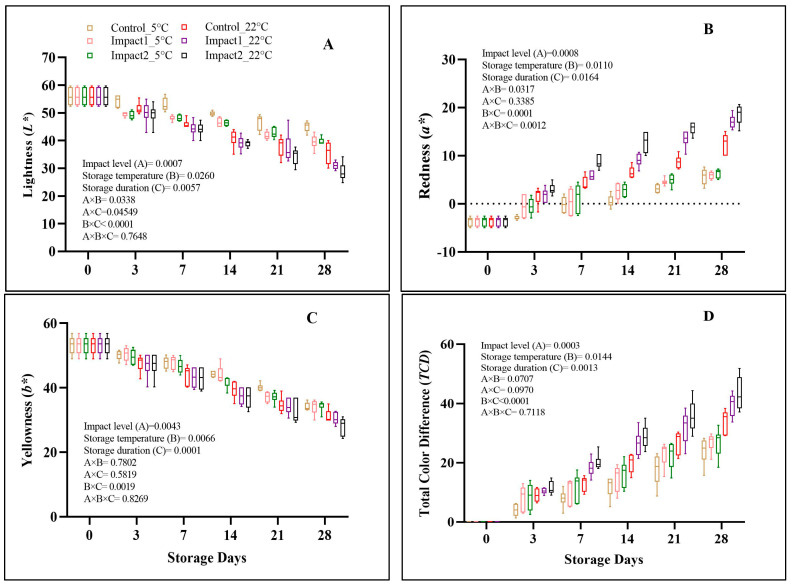
Lightness (L*) (**A**), redness (a*) (**B**), yellowness (b*) (**C**), and total color difference (*TCD*) (**D**) of non-bruised, low- (45°; 1.18 J), and high- (65°; 2.29 J) impact-bruised fruit during 28 days at 5 °C and 22 °C storage conditions. The data are presented using a whisker plot. The total number of readings per day per treatment is 10.

Figure 5 presents the overall changes in color attributes among all pomegranate fruit. There was a significant effect of damage level (*p* = 0.0007), storage temperature (*p* = 0.0260), and storage duration (*p* = 0.0057) on the lightness (L*) of pomegranates (Figure 6A). The L* value reduced gradually across all pomegranate samples at both storage conditions during 28 days of storage. At ambient (22 °C) temperature, the L* value was reduced by 48.72% and 44.54%, on high- and low-impact-bruised pomegranate fruit, respectively, and by 36.20% on the non-bruised fruit (control) on the last d of storage. However, the L* reduction % was 28.94% and 29.28% on low- and high-impact-bruised fruit stored at cold storage conditions (5 °C) after 28 days of storage. The non-bruised pomegranates stored at low temperatures showed the lowest reduction % in the L* value, by 19.32%. The influence of impact bruising, storage temperature, and duration on the redness (a*) of pomegranate fruit was statistically significant (*p* < 0.05), as given in Figure 6B. All fruit samples became redder at the end of the storage period, regardless of the treatments. At the end of 28 days of storage, the a* value increased from −3.63 to 12.5, 17.05, and 18.55 for control, low-, and high-impact-bruised fruit, respectively stored at 22 °C. The changes in a* values were more minor on bruised and non-bruised pomegranate fruit stored at 5 °C compared to those stored at ambient (22 °C) conditions. In addition, the changes in yellowness (b*) were influenced by impact damage (*p* = 0.004345), storage temperature (*p* = 0.0066), and duration (*p* = 0.0001) (Figure 6C).

The lowest b* value was observed after 28 days of storage at ambient (22 °C) storage conditions, with a value of 28.04 for low- and high-impact-bruised fruit. The non-bruised fruit stored at 5 °C recorded fewer changes in yellow color with a value of 34.15 compared to other bruised fruit after 28 days of storage. The differences observed in color values could be attributed to the accumulation and biosynthesis of anthocyanin pigments in the peel of pomegranate fruit, resulting in increasing red coloration, as suggested by Arendayse et al. [31]. They indicated similar results obtained by the current study, where storage at a cold temperature (5 °C) can maintain the peel color of the pomegranate fruit.

Chroma (C*) and hue (H*) values are presented in Table 2. There were significant differences (*p* < 0.05) in C* values and between all investigated factors (impact bruising, storage temperature, and storage duration). The effect of all investigated factors was more pronounced in high- (65°; 2.29 J) impact-bruised fruit which was reduced by 36.75%. Hue (H*), which represents color purity, showed a significant effect with storage duration (*p* = 0.0438) with no further significance of impact damage (*p* = 0.2776) and temperature conditions (*p* = 0.2721). As a result of bruising, the total color change (*TCD*) of pomegranate fruit peel was significantly affected by the level of impact (*p* = 0.0003), storage temperature (*p* = 0.0144), and storage duration (*p* = 0.0013). The *TCD* increased with bruising impact level and storage temperature, reaching the highest values of 34.06, 39.63, and 43.36 after 28 days of storage time for control, low-, and high-impact-damaged pomegranates, stored at 22 °C, respectively. By comparison, pomegranates stored at lower temperature conditions recorded lower values of *TCD* on the last day of storage, which was 24.00 obtained for non-damaged pomegranates and increased with impact levels to 26.84 and 26.95 for low- and high-impact-bruised fruit, respectively. Table 2 shows the mean ± sd values of the browning index (*BI*). Changes in the peel browning index were significantly influenced by bruising and the combination of both storage duration and temperature (*p* < 0.05). After 28 days, fruit stored at 22 °C exhibited an increase in *BI* from the initial of −228.58 (day-0) to 796.52, 1220.67, and 1333.73 for the non-bruised, low- (45°; 1.18 J), and high- (65°; 2.29 J) impact-bruised fruit, respectively. The *BI* increment was slightly lower than that measured after 28 days at a cold temperature (5 °C) for the non-bruised, low-, and high-impact-bruised fruit, with values of 302.07, 353.32, and 378.27, respectively.

Generally, the browning reaction is assumed to be an immediate consequence of the polyphenol oxidase (PPO) and peroxidase (POD) action on polyphenols, which form quinones that produce the browning appearance [34]. These findings revealed that storage temperature is highly linked with the browning associated with the bruise-damaged fruit due to its impact on the polyphenol enzyme activity produced by the fruit [35]. Despite the importance of storage at 5 °C, the slow increase in *BI* observed in fruit at this condition is mainly attributed to the enhancement of some physiological disorders such as chilling and oxidative injuries [36]. Overall, the results presented an excellent relationship between *BI* and *TCD*, therefore suggesting its appropriateness as an essential index for evaluating browning in pomegranates and distinguishing between the non-bruised and bruised fruit. Moreover, Mitsuhashi-Gonzalez et al. [37] found that enzymatic browning in mechanically injured fruit begins by producing phenolic compounds (intra-membrane cell content) in the intercellular space because of cell rupture, which mostly depends on the damage intensity.

#### 3.2.4. Respiration Rate (RR) and Ethylene Production Rate (EPR)

**Figure 7:**
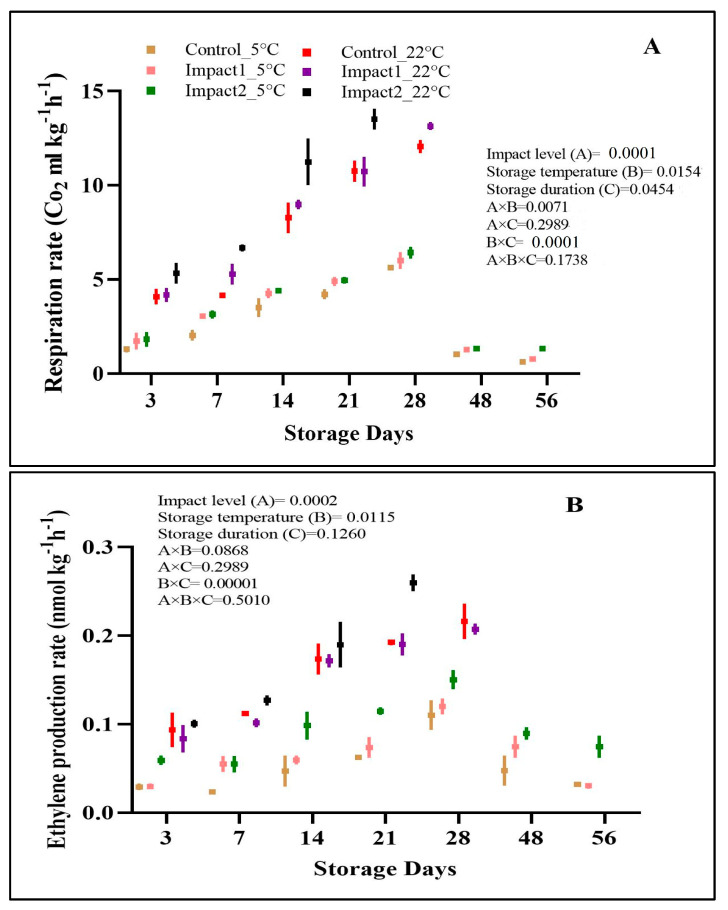
Respiration rate (**A**) and ethylene production rate (**B**) of non-bruised, low—(45°; 1.18 J), and high—(65°; 2.29 J) impact-bruised fruit during 28 days at 5 °C and 22 °C storage conditions. The data were presented using a whisker plot. The total number of readings per day per treatment is 2.

The findings from this study showed that both the respiration rate and ethylene production rate of the examined pomegranates were significantly (*p* < 0.05) dependent on bruising impact level, storage temperature, and storage duration (Figure 7A,B). The *RR* of all samples increased during storage at both storage conditions and was highly enriched in pomegranate fruit stored at ambient (22 °C) storage conditions. The *RR* values reached their peak on day 21 for high-impact-bruised pomegranate fruit (13.49 CO_2_ mL kg^−1^h^−1^) stored at 22 °C and then stopped compared to low-impact bruised fruit and non-bruised fruit. Fawole et al. [1] stated that the anaerobic respiration and metabolic activity that are a consequence of microbial infestation of the fruit could lead to the discontinuation of the *RR* experiment on high-impact-bruised pomegranate fruit.

The non-bruised fruit and low- and high-impact-bruised pomegranates stored at 5 °C showed a slow increment in *RR* followed by a reduction after day 28. Impact level had a considerable influence on the cellular respiration for bruised pomegranate fruit compared to the non-bruised (control) fruit at both storage conditions. This means that at the same storage condition, the damaged fruit respired faster than the control fruit. In addition, the storage temperature of 22 °C increased and showed a five-fold increment in the rate of CO_2_ production in pomegranates compared to those stored at 5 °C. Mechanical damage such as bruising can influence the *RR* of different fresh produce [8]. The present study’s findings support the results recorded by Hussein et al. [9], which stated that pomegranate fruit bruised at higher impact levels showed a two- to three-fold higher *RR* than non-bruised fruit. As observed, the respiration rate reduced with prolonged bruising duration. These results agree with those recorded for citrus [8] and olive [38]. A similar scenario was observed for *EPR* across all pomegranate samples at both storage conditions. Mechanical damage expedited ethylene production mainly at ambient (22 °C) storage conditions. This was also observed in a study conducted on banana fruit by Maia et al. [39].

### 3.3. Multivariate Analysis

#### 3.3.1. Pearson Correlation

The Pearson correlation analysis was conducted to determine the relationships among the physical characteristics of non-bruised (control), low- (45°; 1.18 J), and high- (65°; 2.29 J) impact-damaged fruit during 28 days at 5 °C and 22 °C storage conditions (Table S2). A significant correlation (*, *p* < 0.05; **, *p* < 0.001) was recorded between the majority of the investigated quality attributes (variables) of the pomegranate fruit. In the low- and high-impact bruised fruit at both storage conditions, the *BS* showed a strong positive correlation with *WL%* (*r* ≥ 0.950), a* (*r* ≥ 0.920), *RR (r* ≥ 0.887), and *EPR* (*r* ≥ 0.905). While it exhibited a strong negative correlation with values of firmness (*r* ≥ −0.817), *A_S_* (*r* ≥ −0.940), L*, and b* (*r* ≥ −0.905). A similar scenario was observed with *BA* and *BV* and their correlation with other quality attributes. In all tested fruit across all examined conditions, fruit *WL%* had a significant positive correlation with a* (*r* ≥ 0.967), *TCD* (*r* ≥ 0.981), *BI* (*r* ≥ 0.967), *RR,* and *EPR* (*r* ≥ 0.943). *WL%* was negatively correlated with firmness (*r* ≥ −0.920), *Dg* (*r* ≥ −0.988), L* (*r* ≥ −0.960), and b* (*r* ≥ −0.984). As observed in Table S2, a* strongly correlated with all studied parameters (*p* < 0.001), mainly with *BI* (*r* ≥ −0.999), except in the case of some values with H*. This indicated that increasing the redness of pomegranate fruit due to bruising and storage conditions, particularly at 22 °C, can result from the increment in *BI* over time.

#### 3.3.2. Principal Component Analysis (PCA)

**Figure 8:**
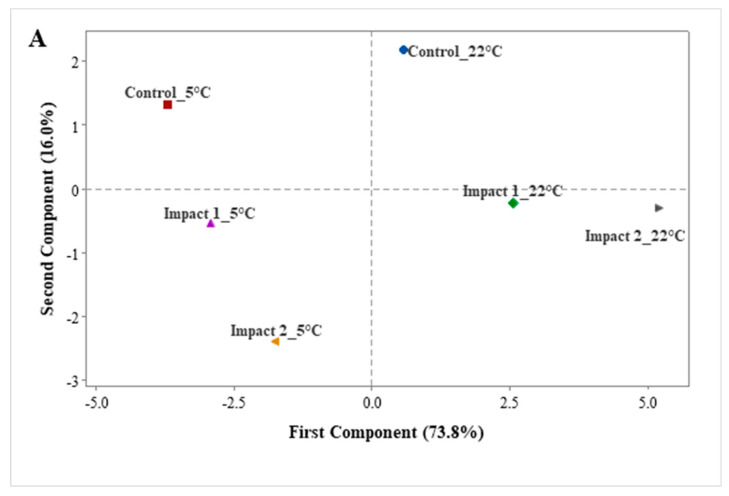

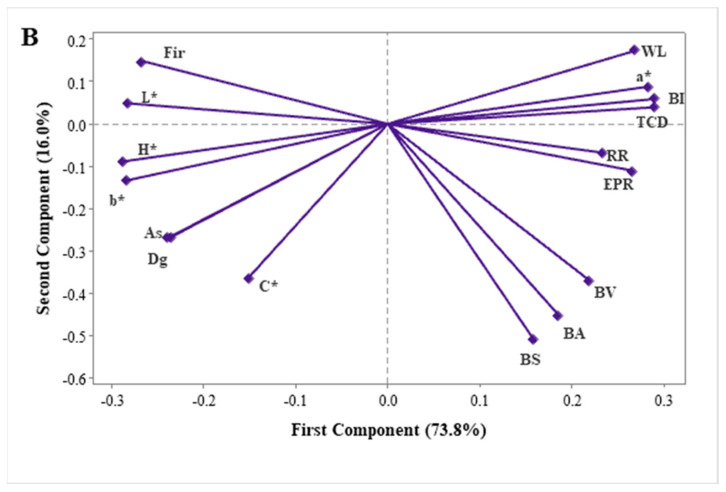
Principal component analysis of the first two components (PC1 and PC2) showing observations (**A**) and variables (**B**) based on the bruise parameters such as bruise area (*BA*), bruise volume (*BV*), and bruise susceptibility (*BS*) and physical attributes such as weight loss % (WL), firmness (Fir), geometric mean diameter (*Dg*), surface area (*A_S_*), lightness (*L*), redness (*a*), yellowness (*b*), the total color difference (*TCD*), chroma (C*), hue (H*), browning index (*BI*), respiration rate (*RR*), and ethylene production rate (*EPR))* of non-bruised, low- (45°; 1.18 J-impact 1), and high- (65°; 2.29 J-impact 2) impact-bruised fruit after 28 days at 5 °C and 22 °C storage conditions.

Principal component analysis (PCA) was implemented to reveal the correlation between the studied quality attributes and bruise parameters with treatment (impact height and storage temperature) of pomegranates during the storage period. Thus, the variability of physical attributes of non-bruised and bruised pomegranates is summarized in principal component analysis (PCA). The location of the impact height and storage temperature after 28 days of storage is demonstrated in Figure 8A, while Figure 8B defines the distribution of quality and bruise parameters by first and second principal component analysis (PCA1 and 2) dimensions. The total variability at the different impact levels and storage conditions was described by 16 principal components (PC1 to PC16), and the first two factors were considered and retained to summarize the pattern of variance among the measured physical variables of the present study. The sum of the two first principal components (PC1 and PC2) explained 89.8% of the variations, with PC1 and PC2 characterizing 73.8% and 16.0%, respectively. Generally, Figure 8A,B shows that the fruit exhibited both distinct and similar variability in features at different impact levels and storage periods.

Figure 8A shows that pomegranate fruit was affected by both studied factors (impact level and storage temperature) after 28 days of storage which recorded a strong correlation with the resulting parameters, respectively. Both storage temperature conditions and impact levels showed comparable characteristics with pomegranate fruit, respectively. Along with PC1, Figure 8B reveals that non-bruised and bruised pomegranate fruit (65°; 2.29 J and 45°; 1.18 J) stored at ambient temperature (22 °C) were more distinctly characterized by higher bruise volume (*BV*), bruise area (*BA*), and bruise susceptibility (*BS*); by respiration rate (*RR*), ethylene production rate (*EPR*), the total color difference (*TCD*), browning index (BI), redness (a*), and weight loss (*WL%*); and by lower firmness (Fir), lightness (L*), hue (H*), yellowness (b*), surface area (*A_s_*), and geometric mean diameter (*Dg*) which were in contrast to those of fruit stored at 5 °C. This could be attributed to the role of low temperature in slowing down the quality changes of pomegranate fruits during storage. Bruise parameters were strongly positively correlated with weight loss%, redness, browning index, and respiration rate, and significantly negatively correlated with firmness, lightness, hue, yellowness, and surface area. This analysis can help to assume changes in quality attributes under prolonged shelf life. The study can finally confirm the importance of storage management to maintain the quality and increase the shelf life of pomegranate fruits during prolonged storage.

## 4. Conclusions

This study has investigated the magnitude of bruise size and the physiological alterations coupled with impact damage and storage in pomegranates. The results showed that bruising measurements (bruise area, bruise volume, and bruise susceptibility) increased as impact level, storage temperature, and storage duration increased. This study showed that storage temperature, storage duration, and impact level had an essential and direct effect on different quality characteristics, mainly fruit color (lightness, redness, yellowness, chroma, total color difference, and browning index), water loss per unit surface area, water loss per unit mass, firmness, geometric mean diameter, respiration rate, and ethylene production rate. An excessive weight loss percentage (20.39%) was detected in high-level impact pomegranate fruit stored at ambient storage conditions on the last d of storage. Additionally, high and low impact levels expedited the peel color changes, mainly at 22 °C in mechanically injured fruit. In addition, there was a significant reduction in the firmness values of pomegranates impacted at high and low levels. The respiration rate and ethylene production rate increased gradually among all studied factors, particularly in bruised and non-bruised fruit stored under ambient conditions. The findings could inform the pomegranate and other fresh fruit industries on the market value reduction and economic loss due to bruising injury-related problems.

## Figures and Tables

**Table 1:** Drop angles used and the resulting impact, rebound, and absorbed energies.

Angles	Rebound Energy (J)	Impacts Energy (J)	Absorbed Energy (J)
(45°)	0.102 ± 0.034	1.292 ± 0.000	1.189 ± 0.109
(65°)	0.248 ± 0.109	2.547 ± 0.000	2.298 ± 0.239

**Table 2:** Chroma (C*), hue (H*), and browning index (*BI*) for non-bruised, low- (45°; 1.18 J), and high- (65°; 2.29 J) impact-bruised fruit during 28 days at 5 °C and 22 °C storage conditions. The values are presented as standard deviation (*SD*) of the mean values ± S.D. of 10 readings of 2 replicates.

Days	Impact Level	Storage Temp. (°C)	C*	H*	*BI*
0	Control	5	53.3 ± 2.78	−1.503 ± 0.018	−228.1 ± 57.39
		22			
	Low	5			
		22			
	High	5			
		22			
3	Control	5	50.22 ± 1.45	−1.51 ± 0.01	−177.3 ± 26.17
		22	47.73 ± 2.66	1.01±1.24	101.40 ± 102.6
	Low	5	50.49 ± 2.11	0.01 ± 1.67	−32.78 ± 156.2
		22	47.18 ± 3.68	1.53 ± 0.02	108.90 ± 82.94
	High	5	49.60 ± 2.26	−0.51 ± 1.59	−38.8 ± 103.7
		22	47.23 ± 3.68	1.50 ± 0.02	198.2 ± 74.13
7	Control	5	47.88 ± 1.91	0.004 ± 1.68	−11.21 ± 97.21
		22	44.25 ± 3.03	1.47 ± 0.02	275.60 ± 98.23
	Low	5	48.01 ± 2.10	−0.06 ± 1.66	29.27 ± 170.8
		22	43.56 ± 3.47	1.43 ± 0.02	393.30 ± 72.64
	High	5	46.83 ± 2.33	0.49 ± 1.56	88.00 ± 175.30
		22	43.82 ± 3.52	1.36 ± 0.04	590.10 ± 95.31
14	Control	5	44.23 ± 0.88	0.51 ± 1.60	28.68 ± 68.74
		22	40.01 ± 2.58	1.40 ± 0.02	430.10 ± 77.02
	Low	5	44.23 ± 2.63	1.51 ± 0.04	161.90 ± 92.9
		22	38.52 ± 2.94	1.33 ± 0.03	606.70 ± 102.20
	High	5	42.06 ± 1.78	1.50 ± 0.03	170.30 ± 70.89
		22	39.24 ± 3.15	1.23 ± 0.06	862.50 ± 139.60
21	Control	5	40.28 ± 1.07	1.49 ± 0.02	182.6 ± 43.31
		22	35.77 ± 2.15	1.32 ± 0.04	556.30 ± 102.7
	Low	5	37.44 ± 1.52	1.44 ± 0.01	273.10 ± 41.45
		22	36.82 ± 2.70	1.19 ± 0.03	914.30 ± 184.90
	High	5	37.46 ± 1.75	1.43 ± 0.03	296.30 ± 75.26
		22	36.15 ± 2.89	1.11 ± 0.06	1070.00 ± 100.50
28	Control	5	35.66 ± 1.01	1.40 ± 0.05	302.1 ± 75.18
		22	33.64 ± 1.82	1.18 ± 0.06	796.50 ± 177.2
	Low	5	34.65 ± 2.28	1.39 ± 0.02	353.30 ± 51.81
		22	35.93 ± 2.05	1.06 ± 0.03	1221.00 ± 137.30
	High	5	35.00 ± 1.74	1.38 ± 0.02	378.30 ± 62.78
		22	33.71 ± 2.09	0.98 ± 0.08	1334.00 ± 151.30
**Level of significance**					
Impact level (*A*)			=0.0382	=0.2776	=0.0011
Storage temperature (*B*)			=0.0213	=0.2721	=0.0144
Storage duration (*C*)			=0.0004	=0.0438	=0.0305
A× B			=0.4766	=0.0627	=0.0274
A× C			=0.9242	=0.0860	=0.3457
B× C			=0.0074	>0.0001	=0.0001
A× B × C			=0.7647	=0.8398	=0.0005

## Data Availability

Data are contained within the article.

## Supplementary Materials

The following supporting information can be downloaded at: https://www.mdpi.com/article/10.3390/foods12061122/s1, Table S1: The reduction % in the pomegranate length (*L*), width (*W*), and thickness (*T*) for non-bruised, low- (45°; 1.18 J), and high- (65°; 2.29 J) impact-bruised fruit during 28 days at 5 °C and 22 °C storage conditions. The values are presented as standard deviation (SD) of the mean values ± S.D. of 2 readings of 2 replicates; Table S2: Pearson correlation coefficients (*r*) between bruise area (*BA*), bruise volume (*BV*), bruise susceptibility (*BS*), weight loss % (WL%), firmness (*Firm*), geometric mean diameter (*Dg*), surface area (*A_S_*), lightness (*L**), redness (*a**), yellowness (b*), the total color difference (*TCD*), chroma (*C**), hue (*H**), browning index (*BI*), respiration rate (*RR*), and ethylene production rate (*EPR*) for non-bruised, low- (45°; 1.18 J), and high- (65°; 2.29 J) impact-bruised fruit during 28 days at 5 °C and 22 °C storage conditions. Significant correlations of two-tailed tests are indicated: *, *p* < 0.05; **, *p* < 0.001. IL: impact level; ST: storage temperature.
