# Supplementary material for: Physiological Response of Stored Pomegranate Fruit Affected by Simulated Impact
Source: Foods. 2023 Mar 7;12(6):1122. doi: 10.3390/foods12061122 (PMC10048388; doi:10.3390/foods12061122)
Supplement: Supplementary file 1 [file foods-12-01122-s001.zip › foods-2246778-supplementary.pdf]

Supplementary material

Table S1. The reduction % in the pomegranate length (L), width (W), and thickness (T) for non-bruised, low (45°; 1.18 J), and high (65°; 2.29 J) impact bruised fruit during 28 days at 5 °C and 22 °C storage conditions. The values are presented as standard deviation (SD) of the mean values ± S.D. of 2 readings of 2 replicates.

Days	Impact level	Storage temp. (°C)	*L* (%)	*W* (%)	*T* (%)
3	Control	5	0.14±0.04	0.22±0.13	0.21±0.20
		22	0.37±0.13	0.30±0.08	0.33±0.22
	Low	5	0.16±0.08	0.21±0.09	0.25±0.10
		22	0.33±0.13	0.42±0.02	0.33±0.18
	High	5	0.25±0.01	0.14±0.02	0.24±0.02
		22	0.42±0.06	0.35±0.06	0.36±0.02
7	Control	5	0.43±0.17	0.59±0.09	0.42±0.40
		22	0.70±0.03	0.55±0.01	0.78±0.21
	Low	5	0.33±0.17	0.35±0.19	0.46±0.02
		22	0.82±0.04	0.81±0.10	0.72±0.22
	High	5	0.43±0.01	0.34±0.14	0.54±0.04
		22	0.95±0.07	0.72±0.27	0.80±0.04
14	Control	5	0.91±0.02	0.95±0.01	0.91±0.33
		22	1.09±0.01	1.00±0.13	1.07±0.11
	Low	5	0.60±0.01	0.60±0.31	0.69±0.02
		22	1.34±0.11	1.11±0.10	1.27±0.18
	High	5	0.72±0.01	0.67±0.13	0.82±0.08
		22	1.46±0.01	1.18±0.20	1.19±0.01
21	Control	5	1.50±0.16	1.52±0.52	1.20±0.59
		22	1.46±0.02	1.38±0.19	1.39±0.16
	Low	5	0.95±0.09	0.92±0.14	0.97±0.10
		22	1.74±0.12	1.51±0.01	1.74±0.16
	High	5	0.96±0.14	0.89±0.06	1.13±0.12
		22	1.91±0.16	1.72±0.28	1.76±0.01
28	Control	5	1.91±0.11	1.91±0.17	1.55±0.58
		22	1.88±0.03	1.89±0.17	1.87±0.1
	Low	5	1.21±0.02	1.18±0.24	1.23±0.25
		22	2.33±0.27	2.04±0.01	2.11±0.04
	High	5	1.24±0.06	1.20±0.08	1.43±0.17
		22	2.57±0.12	2.36±0.45	2.38±0.37
**Level of significance**					
Impact level (A)			= 0.0078	= 0.1235	= 0.0418
Storage temperature (B)			= 0.0149	= 0.0191	=0.0268
Storage duration (C)			= 0.0325	= 0.0115	= 0.0034
A× B			= 0.0304	= 0.0080	= 0.1007
A× C			= 0.9999	= 0.9578	=0.9598
B× C			= 0.0992	= 0.1067	= 0.0057
A× B × C			≤0.0001	= 0.0677	= 0.7070

Table S2. Pearson correlation coefficients (*r*) between bruise area (BA), bruise volume (*BV*), bruise susceptibility (*BS*), weight loss % (*WL%*), firmness (*Firm*), geometric mean diameter (*Dg*), surface area (*As*), lightness (*L**), redness (a*), yellowness (b*), the total color difference (TCD), chroma (*C*), hue (*H*), browning index (*BI*), respiration rate (*RR*), and ethylene production rate (*EPR*) for non-bruised, low (45°; 1.18 J), and high (65°; 2.29 J) impact bruised fruit during 28 days at 5 °C and 22 °C storage conditions. Significant correlations of two-tailed tests are indicated: *, P < 0.05; **, P < 0.001. IL; impact level, ST; storage temperature.

**Quality**	**IL**	**ST**	***BA***	***BV***	***BS***	***WL%***	***Firm***	***Dg***	***As***	***L****	***a****	***b****	***TCD***	***C****	***H****	***BI***	***RR***	***EPR***
BA	Control	5°C	-	-	-	-	-	-	-	-	-	-	-	-	-	-	-	-
		22°C	-	-	-	-	-	-	-	-	-	-	-	-	-	-	-	-
	Low	5°C	1	0.878*	0.882*	0.910*	-0.998**	-0.933**	-0.934**	-0.921**	0.952**	-0.916*	0.930**	-0.920**	0.927**	0.956**	0.977**	0.905*
		22°C	1	0.886*	0.887*	0.972**	-0.977**	-0.977**	-0.978**	-0.981**	0.989**	-0.995**	0.991**	-0.994**	0.644	0.982**	1.000**	0.996**
	High	5°C	1	0.967**	0.966**	0.967**	-0.988**	-0.965**	-0.965**	-0.945**	0.981**	-0.976**	0.972**	-0.980**	0.974**	0.980**	0.988**	0.950**
		22°C	1	0.993**	0.987**	0.965**	-0.970**	-0.964**	-0.965**	-0.982**	0.982**	-0.986**	0.986**	-0.992**	0.643	0.981**	0.991**	0.999**
BV	Control	5°C	-	-	-	-	-	-	-	-	-	-	-	-	-	-	-	-
		22°C	-	-	-	-	-	-	-	-	-	-	-	-	-	-	-	-
	Low	5°C	0.878*	1	1.000**	0.987**	-0.891*	-0.983**	-0.983**	-0.933**	0.951**	-0.976**	0.958**	-0.974**	0.812*	0.943**	0.936**	0.962**
		22°C	0.886*	1	1.000**	0.949**	-0.817*	-0.948**	-0.947**	-0.901*	0.923**	-0.905*	0.908*	-0.848*	0.326	0.938**	0.886*	0.843*
	High	5°C	0.967**	1	1.000**	0.988**	-0.944**	-0.981**	-0.981**	-0.920**	0.954**	-0.986**	0.957**	-0.986**	0.894*	0.946**	0.967**	0.949**
		22°C	0.993**	1	0.997**	0.984**	-0.976**	-0.974**	-0.975**	-0.989**	0.987**	-.995**	0.993**	-0.996**	0.594	0.987**	0.999**	0.994**
BS	Control	5°C	-	-	-	-	-	-	-	-	-	-	-	-	-	-	-	-
		22°C	-	-	-	-	-	-	-	-	-	-	-	-	-	-	-	-
	Low	5°C	0.882*	1.000**	1	0.991**	-0.894*	-0.987**	-0.986**	-0.938**	0.955**	-0.981**	0.963**	-0.979**	0.817*	0.947**	0.937**	0.960**
		22°C	0.887*	1.000**	1	0.950**	-0.817*	-0.947**	-0.946**	-0.900*	0.923**	-0.905*	0.908*	-0.848*	0.326	0.938**	0.887*	0.844*
	High	5°C	0.966**	1.000**	1	0.988**	-0.944**	-0.981**	-0.981**	-0.919**	0.953**	-0.986**	0.957**	-0.986**	0.894*	0.945**	0.967**	0.949**

		22°C	0.987**	0.997**	1	0.992**	-0.962**	-0.983**	-0.983**	-0.989**	0.976**	-0.995**	0.989**	-0.990**	0.540	0.980**	0.993**	0.988**
	Control	5°C	-	-	-	1	-0.994**	-0.988**	-0.988**	-0.977**	0.984**	-0.998**	0.992**	-0.996**	0.901*	0.977**	0.976**	0.995**
		22°C	-	-	-	1	-0.926**	-0.994**	-0.994**	-0.977**	0.965**	-0.990**	0.988**	-0.982**	0.587	0.965**	0.984**	0.943**
WL%	Low	5°C	0.910*	0.987**	0.991**	1	-0.920**	-0.997**	-0.997**	-0.960**	0.974**	-0.998**	0.983**	-0.998**	0.849*	0.967**	0.952**	0.950**
		22°C	0.972**	0.949**	0.950**	1	-0.934**	-0.995**	-0.995**	-0.975**	0.983**	-0.984**	0.981**	-0.959**	0.454	0.986**	0.973**	0.951**
	High	5°C	0.967**	0.988**	0.988**	1	-0.957**	-0.999**	-0.999**	-0.957**	0.979**	-0.998**	0.983**	-0.997**	0.895*	0.974**	0.976**	0.967**
		22°C	0.965**	0.984**	0.992**	1	-0.952**	-0.990**	-0.990**	-0.988**	0.967**	-0.992**	0.984**	-0.979**	0.452	0.974**	0.971**	0.965**
	Control	5°C	-	-	-	-0.994**	1	0.983**	0.983**	0.975**	-0.973**	0.988**	-0.983**	0.987**	-0.890*	-0.965**	-0.958**	-0.983**
		22°C	-	-	-	-0.926**	1	0.950**	0.951**	0.981**	-0.976**	0.963**	-0.952**	0.973**	-0.820*	-0.979**	-0.931**	-0.974**
Firm	Low	5°C	-0.998**	-0.891*	-0.894*	-0.920**	1	0.943**	0.943**	0.939**	-0.966**	0.924**	-0.944**	0.928**	-0.940**	-0.970**	-0.988**	-0.923**
		22°C	-0.977**	-0.817*	-0.817*	-0.934**	1	0.952**	0.953**	0.974**	-0.976**	0.978**	-0.979**	0.990**	-0.700	-0.965**	-0.975**	-0.978**
	High	5°C	-0.988**	-0.944**	-0.944**	-0.957**	1	0.959**	0.959**	0.931**	-0.973**	0.960**	-0.959**	0.963**	-0.980**	-0.975**	-0.972**	-0.934**
		22°C	-0.970**	-0.976**	-0.962**	-0.952**	1	0.947**	0.948**	0.978**	-0.998**	0.976**	-0.986**	0.990**	-0.669	-0.994**	-0.974**	-0.965**
	Control	5°C	-	-	-	-0.988**	0.983**	1	1.000**	0.997**	-0.988**	0.993**	-0.996**	0.994**	-0.951**	-0.988**	-0.987**	-0.974**
		22°C	-	-	-	-0.994**	0.950**	1	1.000**	0.982**	-0.985**	0.996**	-0.997**	0.989**	-0.641	-0.985**	-0.986**	-0.957**
	Low	5°C	-0.933**	-0.983**	-0.987**	-0.997**	0.943**	1	1.000**	0.974**	-0.986**	0.996**	-0.991**	0.996**	-0.876*	-0.982**	-0.972**	-0.964**
Dg		22°C	-0.977**	-0.948**	-0.947**	-0.995**	0.952**	1	1.000**	0.989**	-0.994**	0.991**	-0.992**	0.969**	-0.508	-0.997**	-0.978**	-0.958**
	High	5°C	-0.965**	-0.981**	-0.981**	-0.999**	0.959**	1	1.000**	0.963**	-0.982**	0.996**	-0.986**	0.995**	-0.896*	-0.978**	-0.976**	-0.970**
		22°C	-0.964**	-0.974**	-0.983**	-0.990**	0.947**	1	1.000**	0.993**	-0.965**	0.991**	-0.986**	0.975**	-0.469	-0.976**	-0.979**	-0.983**
	Control	5°C	-	-	-	-0.988**	0.983**	1.000**	1	0.997**	-0.988**	0.993**	-0.996**	0.994**	-0.952**	-0.988**	-0.987**	-0.974**
As		22°C	-	-	-	-0.994**	0.951**	1.000**	1	0.983**	-0.986**	0.996**	-0.997**	0.989**	-0.643	-0.986**	-0.986**	-0.957**
	Low	5°C	-0.934**	-0.983**	-0.986**	-0.997**	0.943**	1.000**	1	0.974**	-0.987**	0.996**	-0.991**	0.996**	-0.876*	-0.982**	-0.972**	-0.963**

		22°C	-0.978**	-0.947**	-0.946**	-0.995**	0.953**	1.000**	1	0.990**	-0.994**	0.992**	-0.993**	0.970**	-0.510	-0.997**	-0.979**	-0.959**
	High	5°C	-0.965**	-0.981**	-0.981**	-0.999**	0.959**	1.000**	1	0.964**	-0.983**	0.996**	-0.986**	0.995**	-0.897*	-0.979**	-0.976**	-0.971**
		22°C	-0.965**	-0.975**	-0.983**	-0.990**	0.948**	1.000**	1	0.993**	-.0966**	0.992**	-0.987**	0.976**	-0.472	-0.977**	-0.979**	-0.983**
	Control	5°C	-	-	-	-0.977**	0.975**	0.997**	0.997**	1	-0.976**	0.985**	-0.989**	0.987**	-0.954**	-0.979**	-0.986**	-0.961**
		22°C	-	-	-	-0.977**	0.981**	0.982**	0.983**	1	-0.979**	0.990**	-0.980**	0.994**	-0.721	-0.982**	-0.969**	-0.977**
*L**	Low	5°C	-0.921**	-0.933**	-0.938**	-0.960**	0.939**	0.974**	0.974**	1	-0.990**	0.965**	-0.995**	0.967**	-0.904*	-0.990**	-0.975**	-0.961**
		22°C	-0.981**	-0.901*	-0.900*	-0.975**	0.974**	0.989**	0.990**	1	-0.992**	0.995**	-0.997**	0.986**	-0.593	-0.992**	-0.983**	-0.974**
	High	5°C	-0.945**	-0.920**	-0.919**	-0.957**	0.931**	0.963**	0.964**	1	-0.988**	0.966**	-0.992**	0.967**	-0.895*	-0.986**	-0.977**	-0.986**
		22°C	-0.982**	-0.989**	-0.989**	-0.988**	0.978**	0.993**	0.993**	1	-0.989**	0.999**	-0.999**	0.994**	-0.559	-0.995**	-0.990**	-0.989**
	Control	5°C	-	-	-	0.984**	-0.973**	-0.988**	-0.988**	-0.976**	1	-0.988**	0.993**	-0.989**	0.951**	0.999**	0.975**	0.970**
		22°C	-	-	-	0.965**	-0.976**	-0.985**	-0.986**	-0.979**	1	-0.990**	0.991**	-0.989**	0.755	1.000**	0.979**	0.981**
*a**	Low	5°C	0.952**	0.951**	0.955**	0.974**	-0.966**	-0.986**	-0.987**	-0.990**	1	-0.976**	0.995**	-0.978**	0.936**	1.000**	0.991**	0.958**
		22°C	0.989**	0.923**	0.923**	0.983**	-0.976**	-0.994**	-0.994**	-0.992**	1	-0.996**	0.998**	-0.983**	0.593	0.999**	0.988**	0.974**
	High	5°C	0.981**	0.954**	0.953**	0.979**	-0.973**	-0.982**	-0.983**	-0.988**	1	-0.987**	0.998**	-0.988**	0.939**	0.999**	0.992**	0.978**
		22°C	0.982**	0.987**	0.976**	0.967**	-0.998**	-0.965**	-0.966**	-0.989**	1	-0.988**	0.995**	-0.997**	0.641	0.999**	0.984**	0.979**
	Control	5°C	-	-	-	-0.998**	0.988**	0.993**	0.993**	0.985**	-0.988**	1	-0.998**	1.000**	-0.918**	-0.984**	-0.988**	-0.992**
		22°C	-	-	-	-0.990**	0.963**	0.996**	0.996**	0.990**	-0.990**	1	-0.998**	0.998**	-0.692	-0.990**	-0.990**	-0.975**
	Low	5°C	-0.916*	-0.976**	-0.981**	-0.998**	0.924**	0.996**	0.996**	0.965**	-0.976**	1	-0.986**	1.000**	-0.853*	-0.969**	-0.952**	-0.941**
		22°C	-0.995**	-0.905*	-0.905*	-0.984**	0.978**	0.991**	0.992**	0.995**	-0.996**	1	-0.999**	0.993**	-0.599	-0.993**	-0.995**	-0.987**
*b**	High	5°C	-0.976**	-0.986**	-0.986**	-0.998**	0.960**	0.996**	0.996**	0.966**	-0.987**	1	-0.990**	1.000**	-0.908*	-0.982**	-0.984**	-0.971**
		22°C	-0.986**	-0.995**	-0.995**	-0.992**	0.976**	0.991**	0.992**	0.999**	-0.988**	1	-0.998**	0.995**	-0.553	-0.993**	-0.993**	-0.992**
TCD	Control	5°C	-	-	-	0.992**	-0.983**	-0.996**	-0.996**	-0.989**	0.993**	-0.998**	1	-0.999**	0.939**	0.992**	0.991**	0.983**

		22°C	-	-	-	0.988**	-0.952**	-0.997**	-0.997**	-0.980**	0.991**	-0.998**	1	-0.993**	0.680	0.991**	0.994**	0.971**
	Low	5°C	0.930**	0.958**	0.963**	0.983**	-0.944**	-0.991**	-0.991**	-0.995**	0.995**	-0.986**	1	-0.988**	0.900*	0.992**	0.977**	0.961**
		22°C	0.991**	0.908*	0.908*	0.981**	-0.979**	-0.992**	-0.993**	-0.997**	0.998**	-0.999**	1	-0.991**	0.604	0.996**	0.992**	0.982**
	High	5°C	0.972**	0.957**	0.957**	0.983**	-0.959**	-0.986**	-0.986**	-0.992**	0.998**	-0.990**	1	-0.991**	0.917**	0.996**	0.990**	0.985**
		22°C	0.986**	0.993**	0.989**	0.984**	-0.986**	-0.986**	-0.987**	-0.999**	0.995**	-0.998**	1	-0.998**	0.589	0.998**	0.991**	0.989**
*C**	Control	5°C	-	-	-	-0.996**	0.987**	0.994**	0.994**	0.987**	-0.989**	1.000**	-0.999**	1	-0.924**	-0.985**	-0.990**	-0.990**
		22°C	-	-	-	-0.982**	0.973**	0.989**	0.989**	0.994**	-0.989**	0.998**	-0.993**	1	-0.726	-0.989**	-0.986**	-0.984**
	Low	5°C	-0.920**	-0.974**	-0.979**	-0.998**	0.928**	0.996**	0.996**	0.967**	-0.978**	1.000**	-0.988**	1	-0.859*	-0.972**	-0.955**	-0.941**
		22°C	-0.994**	-0.848*	-0.848*	-0.959**	0.990**	0.969**	0.970**	0.986**	-0.983**	0.993**	-0.991**	1	-0.667	-0.975**	-0.994**	-0.996**
	High	5°C	-0.980**	-0.986**	-0.986**	-0.997**	0.963**	0.995**	0.995**	0.967**	-0.988**	1.000**	-0.991**	1	-0.914*	-0.984**	-0.986**	-0.972**
		22°C	-0.992**	-0.996**	-0.990**	-0.979**	0.990**	0.975**	0.976**	0.994**	-0.997**	0.995**	-0.998**	1	-0.621	-0.997**	-0.996**	-0.992**
*H**	Control	5°C	-	-	-	0.901*	-0.890*	-0.951**	-0.952**	-0.954**	0.951**	-0.918**	0.939**	-0.924**	1	0.964**	0.937**	0.870*
		22°C	-	-	-	0.587	-0.820*	-0.641	-0.643	-0.721	0.755	-0.692	0.680	-0.726	1	0.757	0.670	0.812*
	Low	5°C	0.927**	0.812*	0.817*	0.849*	-0.940**	-0.876*	-0.876*	-0.904*	0.936**	-0.853*	0.900*	-0.859*	1	0.943**	0.939**	0.833*
		22°C	0.644	0.326	0.326	0.454	-0.700	-0.508	-0.510	-0.593	0.593	-0.599	0.604	-0.667	1	0.564	0.638	0.687
	High	5°C	0.974**	0.894*	0.894*	0.895*	-0.980**	-0.896*	-0.897*	-0.895*	0.939**	-0.908*	0.917**	-0.914*	1	0.943**	0.952**	0.900*
		22°C	0.643	0.594	0.540	0.452	-0.669	-0.469	-0.472	-0.559	0.641	-0.553	0.589	-0.621	1	0.616	0.701	0.701
BI	Control	5°C	-	-	-	0.977**	-0.965**	-0.988**	-0.988**	-0.979**	0.999**	-0.984**	0.992**	-0.985**	0.964**	1	0.977**	0.960**
		22°C	-	-	-	0.965**	-0.979**	-0.985**	-0.986**	-0.982**	1.000**	-0.990**	0.991**	-0.989**	0.757	1	0.978**	0.982**
	Low	5°C	0.956**	0.943**	0.947**	0.967**	-0.970**	-0.982**	-0.982**	-0.990**	1.000**	-0.969**	0.992**	-0.972**	0.943**	1	.993**	0.956**
		22°C	0.982**	0.938**	0.938**	0.986**	-0.965**	-0.997**	-0.997**	-0.992**	0.999**	-0.993**	0.996**	-0.975**	0.564	1	0.982**	0.965**
	High	5°C	0.980**	0.946**	0.945**	0.974**	-0.975**	-0.978**	-0.979**	-0.986**	0.999**	-0.982**	0.996**	-0.984**	0.943**	1	0.989**	0.973**

		22°C	0.981**	0.987**	0.980**	0.974**	-0.994**	-0.976**	-0.977**	-0.995**	0.999**	-0.993**	0.998**	-0.997**	0.616	1	0.983**	0.979**
	Control	5°C	-	-	-	0.976**	-0.958**	-0.987**	-0.987**	-0.986**	0.975**	-0.988**	0.991**	-0.990**	0.937**	0.977**	1	0.972**
		22°C	-	-	-	0.984**	-0.931**	-0.986**	-0.986**	-0.969**	0.979**	-0.990**	0.994**	-0.986**	0.670	0.978**	1	0.973**
RR	Low	5°C	0.977**	0.936**	0.937**	0.952**	-0.988**	-0.972**	-0.972**	-0.975**	0.991**	-0.952**	0.977**	-0.955**	0.939**	0.993**	1	0.965**
		22°C	1.000**	0.886*	0.887*	0.973**	-0.975**	-0.978**	-0.979**	-0.983**	0.988**	-0.995**	0.992**	-0.994**	0.638	0.982**	1	0.996**
	High	5°C	0.988**	0.967**	0.967**	0.976**	-0.972**	-0.976**	-0.976**	-0.977**	0.992**	-0.984**	0.990**	-0.986**	0.952**	0.989**	1	0.986**
		22°C	0.991**	0.999**	0.993**	0.971**	-0.974**	-0.979**	-0.979**	-0.990**	0.984**	-0.993**	0.991**	-0.996**	0.701	0.983**	1	0.994**
	Control	5°C	-	-	-	0.995**	-0.983**	-0.974**	-0.974**	-0.961**	0.970**	-0.992**	0.983**	-0.990**	0.870*	0.960**	0.972**	1
		22°C	-	-	-	0.943**	-0.974**	-0.957**	-0.957**	-0.977**	0.981**	-0.975**	0.971**	-0.984**	0.812*	0.982**	0.973**	1
EPR	Low	5°C	0.905*	0.962**	0.960**	0.950**	-0.923**	-0.964**	-0.963**	-0.961**	0.958**	-0.941**	0.961**	-0.941**	0.833*	0.956**	0.965**	1
		22°C	0.996**	0.843*	0.844*	0.951**	-0.978**	-0.958**	-0.959**	-0.974**	0.974**	-0.987**	0.982**	-0.996**	0.687	0.965**	0.996**	1
	High	5°C	0.950**	0.949**	0.949**	0.967**	-0.934**	-0.970**	-0.971**	-0.986**	0.978**	-0.971**	0.985**	-0.972**	0.900*	0.973**	0.986**	1
		22°C	0.999**	0.994**	0.988**	0.965**	-0.965**	-0.983**	-0.983**	-0.989**	0.979**	-0.992**	0.989**	-0.992**	0.701	0.979**	0.994**	1
